# Bis(4-methyl­piperidinium) hexa­chlorido­stannate(IV)

**DOI:** 10.1107/S1600536808007368

**Published:** 2008-03-29

**Authors:** Saira Shahzadi, Hamid Nawaz Khan, Saqib Ali, Madeleine Helliwell

**Affiliations:** a Department of Chemistry, GC University, Faisalabad, Pakistan; b Department of Chemistry, Quaid-i-Azam University, Islamabad 45320, Pakistan; c School of Chemistry, The University of Manchester, Manchester M13 9PL, England

## Abstract

The crystal structure of the title compound, (C_6_H_14_N)_2_[SnCl_6_], is built of 4-methyl­piperidinium cations, occupying special positions on the mirror plane, and hexa­chloridostannate(IV) anions on a special position of 2/*m* symmetry. The ions are linked *via* N—H⋯Cl hydrogen bonds into chains running along the *b* axis.

## Related literature

For related literature, see: Shahzadi, Ali & Fettouhi (2006 ▶); Shahzadi, Ali, Bhatti *et al.* (2006 ▶).
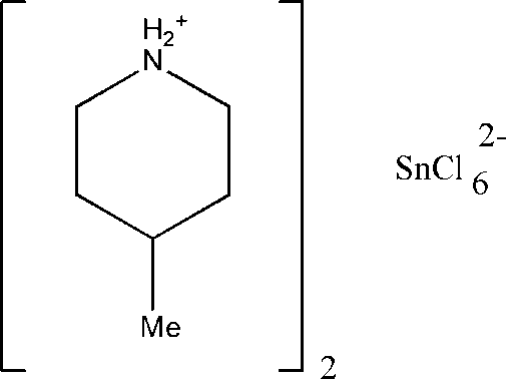


## Experimental

### 

#### Crystal data

(C_6_H_14_N)_2_[SnCl_6_]
*M* *_r_* = 531.75
Orthorhombic, 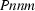

*a* = 13.123 (5) Å
*b* = 7.722 (5) Å
*c* = 10.500 (5) Å
*V* = 1064.0 (9) Å^3^
*Z* = 2
Mo *K*α radiation
μ = 1.95 mm^−1^
*T* = 100 (2) K
0.25 × 0.25 × 0.25 mm

#### Data collection

Bruker APEX CCD area-detector diffractometer
Absorption correction: none
7975 measured reflections
1153 independent reflections
1055 reflections with *I* > 2σ(*I*)
*R*_int_ = 0.050

#### Refinement

*R*[*F* ^2^ > 2σ(*F* ^2^)] = 0.018
*wR*(*F* ^2^) = 0.040
*S* = 1.04
1153 reflections
65 parameters
H atoms treated by a mixture of independent and constrained refinement
Δρ_max_ = 0.78 e Å^−3^
Δρ_min_ = −0.29 e Å^−3^

### 

Data collection: *SMART* (Bruker, 2001 ▶); cell refinement: *SAINT* (Bruker, 2002 ▶); data reduction: *SAINT*; program(s) used to solve structure: *SHELXS97* (Sheldrick, 2008 ▶); program(s) used to refine structure: *SHELXL97* (Sheldrick, 2008 ▶); molecular graphics: *SHELXTL* (Sheldrick, 2008 ▶) and *PLATON* (Spek, 2003 ▶); software used to prepare material for publication: *SHELXTL*.

## Supplementary Material

Crystal structure: contains datablocks global, I. DOI: 10.1107/S1600536808007368/ya2069sup1.cif

Structure factors: contains datablocks I. DOI: 10.1107/S1600536808007368/ya2069Isup2.hkl

Additional supplementary materials:  crystallographic information; 3D view; checkCIF report

## Figures and Tables

**Table 1:** Hydrogen-bond geometry (Å, °)

*D*—H⋯*A*	*D*—H	H⋯*A*	*D*⋯*A*	*D*—H⋯*A*
N1—H1N⋯Cl1	0.88 (3)	2.63 (3)	3.258 (3)	129 (2)
N1—H2N⋯Cl2^i^	0.84 (3)	2.72 (2)	3.413 (2)	141.6 (5)
N1—H2N⋯Cl2^ii^	0.84 (3)	2.72 (2)	3.413 (2)	141.6 (5)

Symmetry codes: (i) 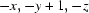; (ii) 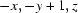.

**Fig. 1.**
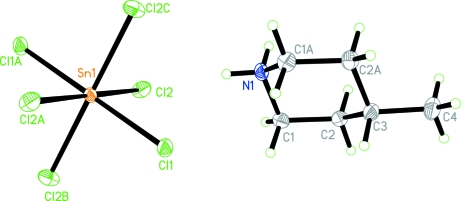
Structure of (I) with displacement ellipsoids drawn at the 50% probability level. The unlabelled atoms of the 4-methylpiperidinium cation are symmetry related (symmetry code x, y, -z). The unlabelled Cl atoms are symmetry related to Cl1 (symmetry code -x, 2 - y, -z) and Cl2 (symmetry codes -x, 2 - y, -z; -x, 2 - y, z; x, y, -z).

**Fig. 2.**
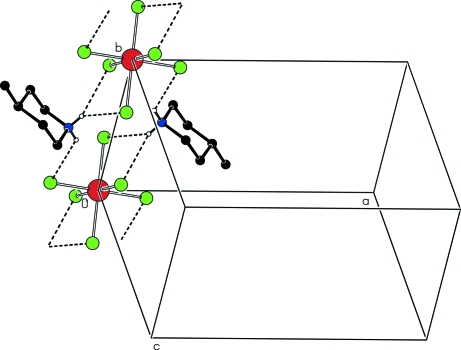
Fragment of the crystal packing of (I) showing chain along the b axis, viewed approximately along the diagonal of the bc-plane; H-bonds are shown as dashed lines.

## supplementary crystallographic information

### Comment

We report here the crystal structure of the title compound (I) as shown in Fig. 1. The Sn1—Cl distances span the range of 2.417 (1)–2.431 (1) Å; the N1—C1 bond is 1.500 (2) Å. The N—H···Cl bonds link the ions into chains along the *b* axis (Table 1, Fig. 2).

### Experimental

The 4-methyl-1-piperidine carbodithioic acid (3.0 g, 17.1 mmol) and tin tetrachloride pentahydrate (5.99 g, 17.1 mmol) were added to 100 ml of dry methanol in round bottom flask and stirred for 6 h. The resulting clear solution was evaporated at room temperature. Colourless crystals of the title compound were obtained after recrystallization in chlorofom and n-hexane (1:1). Yield: 64%. m.p. 228°C.

### Refinement

H atoms bonded to C1—C3 were included in riding motion approximation in calculated positions with C—H distances of 0.99 Å and *U*_iso_ 1.2 times those of the parent atoms; those bonded to C4 and N1 were located in a difference Fourier map and refined isotropically with *U*_iso_ 1.2 times those of the parent atoms (C4 - H distances 0.93 (2) and 0.95 (3) Å and N1 - H 0.84 (3) and 0.88 (3) Å).

### Crystal data

**Table:**

(C_6_H_14_N)_2_[SnCl_6_]	*F*_000_ = 532
*M**_r_* = 531.75	*D*_x_ = 1.660 Mg m^−^^3^
Orthorhombic, *P**n**n**m*	Mo *K*α radiation λ = 0.71069 Å
Hall symbol: -P22n	Cell parameters from 3718 reflections
*a* = 13.123 (5) Å	θ = 2.5–26.3º
*b* = 7.722 (5) Å	µ = 1.95 mm^−^^1^
*c* = 10.500 (5) Å	*T* = 100 (2) K
*V* = 1064.0 (9) Å^3^	Pyramidal, colourless
*Z* = 2	0.25 × 0.25 × 0.25 mm

### Data collection

**Table:**

Bruker APEX CCD area-detector diffractometer	1055 reflections with *I* > 2σ(*I*)
Radiation source: fine-focus sealed tube	*R*_int_ = 0.050
Monochromator: graphite	θ_max_ = 26.3º
*T* = 100(2) K	θ_min_ = 2.5º
φ and ω scans	*h* = −16→16
Absorption correction: none	*k* = −9→9
7975 measured reflections	*l* = −13→13
1153 independent reflections	

### Refinement

**Table:**

Refinement on *F*^2^	Secondary atom site location: difference Fourier map
Least-squares matrix: full	Hydrogen site location: inferred from neighbouring sites
*R*[*F*^2^ > 2σ(*F*^2^)] = 0.018	H atoms treated by a mixture of independent and constrained refinement
*wR*(*F*^2^) = 0.040	*w* = 1/[σ^2^(*F*_o_^2^) + (0.0164*P*)^2^] where *P* = (*F*_o_^2^ + 2*F*_c_^2^)/3
*S* = 1.04	(Δ/σ)_max_ < 0.001
1153 reflections	Δρ_max_ = 0.78 e Å^−^^3^
65 parameters	Δρ_min_ = −0.29 e Å^−^^3^
Primary atom site location: structure-invariant direct methods	Extinction correction: none

### Special details

**Table:**

Geometry. All e.s.d.'s (except the e.s.d. in the dihedral angle between two l.s. planes) are estimated using the full covariance matrix. The cell e.s.d.'s are taken into account individually in the estimation of e.s.d.'s in distances, angles and torsion angles; correlations between e.s.d.'s in cell parameters are only used when they are defined by crystal symmetry. An approximate (isotropic) treatment of cell e.s.d.'s is used for estimating e.s.d.'s involving l.s. planes.
Refinement. Refinement of *F*^2^ against ALL reflections. The weighted *R*-factor *wR* and goodness of fit *S* are based on *F*^2^, conventional *R*-factors *R* are based on *F*, with *F* set to zero for negative *F*^2^. The threshold expression of *F*^2^ > σ(*F*^2^) is used only for calculating *R*-factors(gt) *etc*. and is not relevant to the choice of reflections for refinement. *R*-factors based on *F*^2^ are statistically about twice as large as those based on *F*, and *R*- factors based on ALL data will be even larger.

### Fractional atomic coordinates and isotropic or equivalent isotropic displacement parameters (Å^2^)

**Table:**

	*x*	*y*	*z*	*U*_iso_*/*U*_eq_	
Sn1	0.0000	1.0000	0.0000	0.01428 (9)	
Cl1	0.18119 (5)	0.94377 (8)	0.0000	0.02127 (15)	
Cl2	−0.02412 (3)	0.77582 (6)	0.15973 (4)	0.02279 (12)	
N1	0.16716 (17)	0.5226 (3)	0.0000	0.0187 (5)	
H1N	0.128 (2)	0.615 (4)	0.0000	0.022*	
H2N	0.130 (2)	0.434 (4)	0.0000	0.022*	
C1	0.23016 (14)	0.5228 (2)	0.11919 (17)	0.0202 (4)	
H1C	0.1850	0.5171	0.1946	0.024*	
H1D	0.2701	0.6313	0.1242	0.024*	
C2	0.30167 (13)	0.3686 (2)	0.11902 (17)	0.0194 (4)	
H2A	0.2611	0.2607	0.1235	0.023*	
H2B	0.3456	0.3736	0.1956	0.023*	
C3	0.36885 (19)	0.3640 (3)	0.0000	0.0194 (6)	
H3	0.4130	0.4696	0.0000	0.023*	
C4	0.4374 (2)	0.2055 (4)	0.0000	0.0271 (7)	
H4A	0.4780 (15)	0.200 (3)	−0.0726 (19)	0.032*	
H4B	0.399 (2)	0.102 (4)	0.0000	0.032*	

### Atomic displacement parameters (Å^2^)

**Table:**

	*U*^11^	*U*^22^	*U*^33^	*U*^12^	*U*^13^	*U*^23^
Sn1	0.01250 (13)	0.01126 (13)	0.01907 (14)	−0.00031 (9)	0.000	0.000
Cl1	0.0136 (3)	0.0153 (3)	0.0349 (4)	0.0005 (2)	0.000	0.000
Cl2	0.0214 (2)	0.0228 (2)	0.0242 (3)	−0.00568 (18)	−0.00453 (18)	0.00756 (19)
N1	0.0165 (11)	0.0122 (12)	0.0274 (13)	0.0012 (9)	0.000	0.000
C1	0.0205 (10)	0.0199 (10)	0.0203 (10)	−0.0007 (8)	−0.0011 (7)	−0.0040 (8)
C2	0.0189 (10)	0.0200 (10)	0.0192 (10)	0.0010 (8)	−0.0028 (8)	−0.0011 (8)
C3	0.0151 (13)	0.0189 (14)	0.0241 (15)	0.0003 (10)	0.000	0.000
C4	0.0239 (16)	0.0321 (18)	0.0252 (17)	0.0102 (13)	0.000	0.000

### Geometric parameters (Å, °)

**Table:**

Sn1—Cl1	2.4170 (11)	C1—H1C	0.9900
Sn1—Cl1^i^	2.4170 (11)	C1—H1D	0.9900
Sn1—Cl2^ii^	2.4310 (11)	C2—C3	1.530 (2)
Sn1—Cl2^iii^	2.4310 (11)	C2—H2A	0.9900
Sn1—Cl2^i^	2.4310 (11)	C2—H2B	0.9900
Sn1—Cl2	2.4310 (11)	C3—C4	1.519 (4)
N1—C1^iii^	1.500 (2)	C3—C2^iii^	1.530 (2)
N1—C1	1.500 (2)	C3—H3	1.0000
N1—H1N	0.88 (3)	C4—H4A	0.931 (19)
N1—H2N	0.84 (3)	C4—H4B	0.95 (3)
C1—C2	1.516 (2)		
			
Cl1—Sn1—Cl1^i^	180.000 (5)	N1—C1—C2	109.83 (15)
Cl1—Sn1—Cl2^ii^	89.990 (19)	N1—C1—H1C	109.7
Cl1^i^—Sn1—Cl2^ii^	90.010 (19)	C2—C1—H1C	109.7
Cl1—Sn1—Cl2^iii^	90.010 (19)	N1—C1—H1D	109.7
Cl1^i^—Sn1—Cl2^iii^	89.990 (19)	C2—C1—H1D	109.7
Cl2^ii^—Sn1—Cl2^iii^	180.0	H1C—C1—H1D	108.2
Cl1—Sn1—Cl2^i^	89.990 (19)	C1—C2—C3	112.08 (16)
Cl1^i^—Sn1—Cl2^i^	90.010 (19)	C1—C2—H2A	109.2
Cl2^ii^—Sn1—Cl2^i^	87.24 (5)	C3—C2—H2A	109.2
Cl2^iii^—Sn1—Cl2^i^	92.76 (5)	C1—C2—H2B	109.2
Cl1—Sn1—Cl2	90.010 (19)	C3—C2—H2B	109.2
Cl1^i^—Sn1—Cl2	89.990 (19)	H2A—C2—H2B	107.9
Cl2^ii^—Sn1—Cl2	92.76 (5)	C4—C3—C2^iii^	111.10 (15)
Cl2^iii^—Sn1—Cl2	87.24 (5)	C4—C3—C2	111.10 (15)
Cl2^i^—Sn1—Cl2	180.0	C2^iii^—C3—C2	109.6 (2)
C1^iii^—N1—C1	113.1 (2)	C4—C3—H3	108.3
C1^iii^—N1—H1N	108.7 (8)	C2^iii^—C3—H3	108.3
C1—N1—H1N	108.7 (8)	C2—C3—H3	108.3
C1^iii^—N1—H2N	108.6 (10)	C3—C4—H4A	112.1 (14)
C1—N1—H2N	108.6 (10)	C3—C4—H4B	111.2 (18)
H1N—N1—H2N	109 (3)	H4A—C4—H4B	105.6 (17)
			
C1^iii^—N1—C1—C2	−56.8 (2)	C1—C2—C3—C4	−178.20 (18)
N1—C1—C2—C3	55.7 (2)	C1—C2—C3—C2^iii^	−55.1 (2)

Symmetry codes: (i) −*x*, −*y*+2, −*z*; (ii) −*x*, −*y*+2, *z*; (iii) *x*, *y*, −*z*.

### Hydrogen-bond geometry (Å, °)

**Table:**

*D*—H···*A*	*D*—H	H···*A*	*D*···*A*	*D*—H···*A*
N1—H1N···Cl1	0.88 (3)	2.63 (3)	3.258 (3)	129 (2)
N1—H2N···Cl2^iv^	0.84 (3)	2.72 (2)	3.413 (2)	141.6 (5)
N1—H2N···Cl2^v^	0.84 (3)	2.72 (2)	3.413 (2)	141.6 (5)

Symmetry codes: (iv) −*x*, −*y*+1, −*z*; (v) −*x*, −*y*+1, *z*.
